# α_v_β_3_-Specific Gold Nanoparticles for Fluorescence Imaging of Tumor Angiogenesis

**DOI:** 10.3390/nano11010138

**Published:** 2021-01-08

**Authors:** Marc Pretze, Valeska von Kiedrowski, Roswitha Runge, Robert Freudenberg, René Hübner, Güllü Davarci, Ralf Schirrmacher, Carmen Wängler, Björn Wängler

**Affiliations:** 1 Department of Nuclear Medicine, University Hospital Carl Gustav Carus, TU Dresden, 01307 Dresden, Germany; Roswitha.Runge@uniklinikum-dresden.de (R.R.); Robert.Freudenberg@uniklinikum-dresden.de (R.F.); 2 Molecular Imaging and Radiochemistry, Department of Clinical Radiology and Nuclear Medicine, Medical Faculty Mannheim of Heidelberg University, 68167 Mannheim, Germany; Valeska.vonKiedrowski@medma.uni-heidelberg.de (V.v.K.); Guellue.Davarci@medma.uni-heidelberg.de (G.D.); Bjoern.Waengler@medma.uni-heidelberg.de (B.W.); 3 Helmholtz-Zentrum Dresden-Rossendorf, Institute of Ion Beam Physics and Materials Research, 01328 Dresden, Germany; r.huebner@hzdr.de; 4 Department of Oncology, Division of Oncological Imaging, University of Alberta, Edmonton, AB T6G 2R3, Canada; schirrma@ualberta.ca; 5 Biomedical Chemistry, Department of Clinical Radiology and Nuclear Medicine, Medical Faculty Mannheim of Heidelberg University, 68167 Mannheim, Germany; Carmen.Waengler@medma.uni-heidelberg.de

**Keywords:** gold nanoparticle, optical imaging, radiosensitizer, tumor angiogenesis, RGD peptide

## Abstract

This paper reports on the development of tumor-specific gold nanoparticles (AuNPs) as theranostic tools intended for target accumulation and the detection of tumor angiogenesis via optical imaging (OI) before therapy is performed, being initiated via an external X-ray irradiation source. The AuNPs were decorated with a near-infrared dye, and RGD peptides as the tumor targeting vector for α_v_β_3_-integrin, which is overexpressed in tissue with high tumor angiogenesis. The AuNPs were evaluated in an optical imaging setting in vitro and in vivo exhibiting favorable diagnostic properties with regards to tumor cell accumulation, biodistribution, and clearance. Furthermore, the therapeutic properties of the AuNPs were evaluated in vitro on pUC19 DNA and on A431 cells concerning acute and long-term toxicity, indicating that these AuNPs could be useful as radiosensitizers in therapeutic concepts in the future.

## 1. Introduction

In recent years, gold nanoparticles (AuNPs) have gained serious attention since their first use as radioactive ^198^Au-nanocolloid in the early 1950s for nanobrachytherapy [1,2,3]. Since then, the focus has shifted to the development of ultra-small target-specific AuNPs with a very narrow size distribution and, ultimately, tailored shapes for use in various imaging modalities such as CT [4], Raman [5], or photoacoustic imaging [6]. On the one hand AuNPs represent a perfect platform for multimerization of target-specific effectors on their surface and on the other hand they offer the possibility of detection using multimodal imaging techniques by surface modification [7], as well as for theranostic purposes [8,9,10,11]. Many approaches of AuNPs with a size of >10 nm are based on a phenomenon typically known as ‘enhanced permeability and retention’ (EPR) effect due to passive extravasation of nanoparticles across the perforated vasculature of tumors [12]. Rapid renal clearance is preferable for radioactive diagnostic nanoparticles to avoid a high radiation burden on healthy organs and tissues, which can be achieved for AuNPs smaller than 6 nm in diameter [13]. The development of methods for the synthesis of ultrasmall (<5 nm) AuNPs [14] followed by surface-modification for enhanced stability and homogenization [15,16,17] paved the way for functionalization [18]. The high affinity of sulfur for gold surfaces and the formation of stable and covalent Au-S bonds [19] enable a fast and facile functionalization of AuNPs with thiol-modified (bio-)molecules. Furthermore, PEGylation of the AuNPs leads to a higher bioavailability, as it hinders in vivo formation of a protein corona around the AuNPs [20,21]. Therefore, ultra-small target-specific AuNPs can be developed and functionalized with small molecules [11], antibodies [22], peptides [23], and natural products [24,25]. For molecular imaging, AuNPs can be functionalized with near-infrared dyes [26,27], with radionuclides like fluorine-18, copper-64 or gallium-68 for positron emission tomography (PET) [28,29,30,31,32], and with Magnetic Resonance Imaging (MRI)-relevant metals like gadolinium [33,34]. Additionally, their therapeutic application [35], and particularly their ability to be applicable as a radiosensitizer by Auger-Meitner electron (AME) emission induced by external gamma activation [36,37,38] or β^-^ emission of 412 keV electrons induced by neutron activation of natural ^197^Au generating [^198^Au]AuNPs [23,25,39,40,41] are of special interest.

The focus of this work is based on the development of theragnostic agents using targeted gold nanoparticles for near-infrared (NIR) fluorescent optical imaging (OI) [42]. Surface-functionalization was easily achieved by ligand exchange, introducing, in addition to different reporter or targeting moieties, functional groups for further chemoselective conjugation to which complementary functionalized targeting vectors are then attached [43]. The RGD peptide motif is known to bind to the transmembrane α_v_β_3_ integrin, which is overexpressed during tumor angiogenesis for example on glioma (U87MG) or on epidermoid (A431) cells [44,45,46,47]. To achieve target-specific accumulation in tissue with high tumor angiogenesis, the AuNPs were functionalized with a c(RGDfK) derivative [41,48].

## 2. Materials and Methods

General procedures. All reagents and solvents were purchased from commercial suppliers and were used without further purification. NIR-dye (SIDAG precursor [49]) was purchased from Chess, Mannheim, Germany. NMR spectra were recorded on a 300 MHz Varian Mercury Plus or a 500 MHz Varian NMR System spectrometer (Palo Alto, CA, USA). Chemical shifts (*δ*) are given in ppm and are relative to (CH_3_)_4_Si (^1^H, ^13^C). Mass spectra were obtained on a Bruker Daltonics microflex MALDI-TOF mass spectrometer (Bremen, Germany). Preparative column chromatography was performed on Merck silica gel 60. Reactions were monitored by thin-layer chromatography (TLC) on Merck silica gel F254 aluminum plates, with visualization under UV (λ = 254 nm) or by evaluation using ninhydrin and heating. If necessary, the purity was determined by high performance liquid chromatography (HPLC). The purity of all final compounds was 95% or higher. HPLC was performed on a Dionex UltiMate 3000 HPLC system (Thermo Scientific, Dreieich, Germany), equipped with a reverse phase column (Analytical: Merck Chromolith RP-18e; 100 × 4.6 mm plus a guard column 5 × 4.6 mm; semipreparative: Chromolith RP-18e; 100 × 10 mm plus a guard column 10 × 4.6 mm), and a UV-diode array detector (210 nm, 254 nm). The solvent system used was a gradient of acetonitrile:water (containing 0.1% TFA) (0–5 min: 0–100% MeCN) at a flow rate of 4 mL/min unless otherwise stated. The purification of AuNPs was performed via dialysis (molecular weight cut-off of 14,000 g/mol, Visking, Roth) against distilled water and via size-exclusion chromatography using Sephadex G25 PD10 columns and distilled water as eluent. The purity of the AuNPs was verified by size exclusion HPLC using a Phenomenex PolySep™-SEC GFC-P 4000, LC Column 300 × 7.8 mm and a 35 mm PolySep guard column with water (0.8 mL/min) as eluent on a Thermofisher Ulti HPLC system. Irradiation experiments were performed in an X-ray chamber on a Maxishot Y.TU 320-D03 (Yxlon, Hamburg, Germany) (200 kV, 20 mA, dose rate *D* = 1.24 Gy/min). Re-188-solution was obtained by elution with 0.9% NaCl from an ^188^W/^188^Re-generator (OncoBeta, Garching, Germany). The eluat was concentrated by a QMA cartridge (WAT023525, Waters, Eschborn, Germany), and eventually eluted W-188 was trapped on an alumina cartridge (WAT023561, Waters).

Thiol-PEG_3_-OH and furan-protected thiol-PEG_4_-maleimide [50] were synthesized according to Zhu et al. [42] without any modification. A brief description of the AuNP syntheses can be found in the supporting information.

Affinity experiments. The α_v_β_3_-binding affinities of the RGD peptides and the respectiviely modified AuNPs were determined on A431 tumor cells by in vitro competitive displacement experiments. A431 cells were seeded onto 24-well plates 2–3 days prior to assay start to reach 0.4–0.5 × 10^6^ cells per well. A special binding buffer (Tris·HCl 25 mM, NaCl 150 mM, CaCl_2_ 1 mM, MgCl_2_ 0.5 mM, MnCl_2_ 1 mM, pH 7.4, BSA 0.5%) was used for incubation with 0.13 nM ^125^I-Echistatin (81.4 GBq/μmol) as the α_v_β_3_-specific radioligand in the presence of increasing concentrations (0–100 μM) of competing c(RGDfK) peptide or c(RGDfK)-modified AuNPs (0–30 µM). The IC_50_ values were obtained using the software Origin v9.6.0.172 (Nonlinear Fit, Growth/Sigmoidal, DoseResp, Levenberg Marquardt Fit).

Fluorescence microscopy on Leica TCS SP8. For fluorescence microscopy, cells were seeded onto coverslips for more than 2 days, then washed with PBS and incubated for 1, 3, 6, and 24 h at 37 °C in 5% CO_2_ with the respective media containing AuNPs (100 µg/mL, 0.33 µM). For blocking experiments, c(RGDfK) (150 µg/mL, 0.25 mM) was added to the wells together with the AuNPs. Afterwards, the cells were washed with PBS and incubated with CellMask Orange-solution (1 × working solution) for 15 min at 37 °C. Cells were fixed with 1:1 medium: 4% formaldehyde in PBS for 2 min at ambient temperature and then with 4% formaldehyde in PBS for 15 min at ambient temperature. Cells were then washed 3 × with PBS, and coverslips were prepared onto an object plate with Sytox Green-solution (8.3 µM, 10 µL). Fluorescence microscopy was performed on a Leica TCS SP8 confocal microscope with lasers at λ = 488, 552 and 638 nm. Overlays of microscopies were generated using the FIJI software (v1.50e).

Colony formation assay. Three days prior to the experiments, 150,000 cells were seeded into a 6-well plate. A431 cells were incubated for 24 h in the presence or absence of the α_v_β_3_-specific or non-specific AuNPs. After incubation in the presence or absence of the AuNPs, the cells were washed, and the cell medium was refreshed. Subsequently, the cells were irradiated with X-rays (Maxishot Y.TU, Yxlon, Hamburg) up to 12 min to reach a maximum dose of 10 Gy. After irradiation, the cells were harvested, and a colony formation assay was performed in triplicate for each irradiation point with 1000 cells per well in a 6-well plate. Colonies were cultivated in cell medium for 7 days and then washed with 1 mL PBS, fixed with 2 mL 80% ethanol for 10 min and incubated with 2 mL crystal violet dye solution for 30 min. Afterwards, colonies were washed with distilled water, dried, and counted by light microscopy. Colonies of more than 50 cells were deemed to be survivors, and the plating efficiency for each sample was estimated based on the initial number of seeded cells. The clonogenic cell survival was calculated as the relative plating efficiency of treated vs. untreated samples. Triplicate samples were prepared for each treatment and experimental condition.

In vivo experiments. The in vivo proof-of-concept was performed using male athymic nude mice (Rj:ATHYM-*Foxn1^nu/nu^*) obtained from Janvier Labs. 5 × 10^6^ A431 cells (100 µL, PBS with or without matrigel:PBS v:v = 1:1, unsieved) were inoculated subcutaneously in the left thigh when the mice were 57 weeks old. Mouse health and tumor growth were checked daily until the tumor reached a diameter of 2–5 mm (3–6 weeks for A431). Tumor growth was slower for the matrigel injection and the tumor was smaller. After the tumors reached a sufficient size for imaging, the AuNPs were injected intravenously into the tail vein and their distribution in vivo was monitored after 1, 2, 4, 24, 28, 48, and 72 h via optical imaging (excitation 730 nm, emission 790 nm, 60 s) followed by X-ray imaging (0.8 mm filter, 45 kV, 5 s) (In Vivo Xtreme, Bruker, Ettlingen). After the last time point, animals were sacrificed, the organs were harvested, and measured ex vivo with the In Vivo Xtreme system. The region of interest (ROI) was drawn by hand on the organs for calculation of the uptake of the AuNPs in the respective organs. Amide (v1.0.4) was used for the fusion of the images. All injections and measurements with mice were performed under anesthesia (2–3% isoflurane/O_2_, 2–3 mL/min).

## 3. Results

### 3.1. Synthesis and Functionalization of Gold Nanoparticles

**Figure 1:**
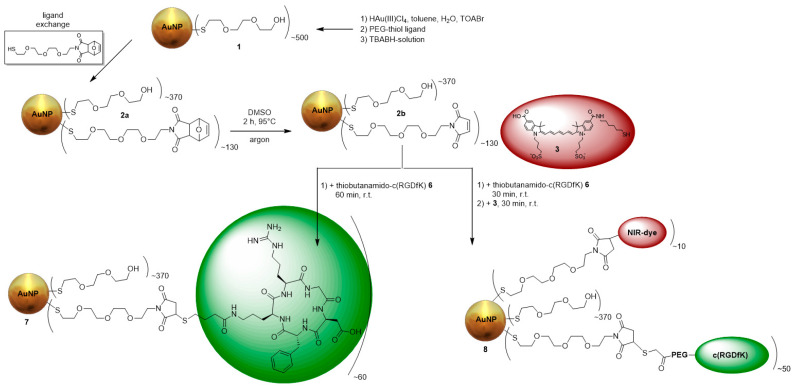
Synthesis of the different RGD-functionalized AuNPs **7** and **8**.

**Figure A1:**
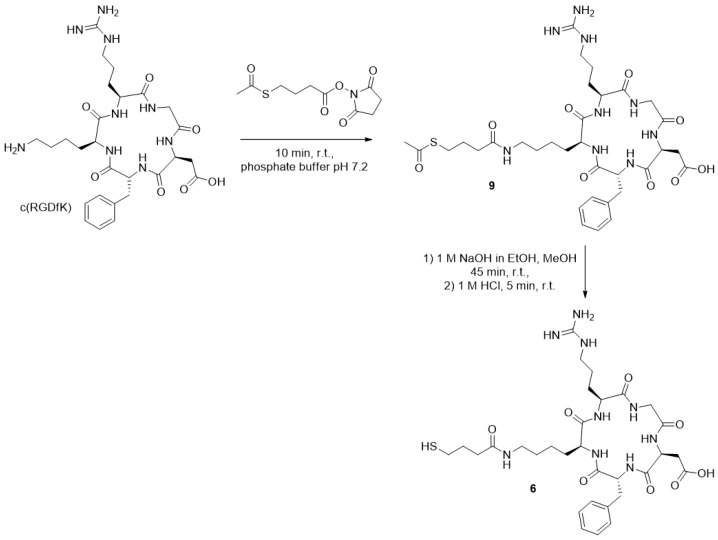
Synthesis of thiol-modified c(RGDfK) **6**.

**Figure A2:**
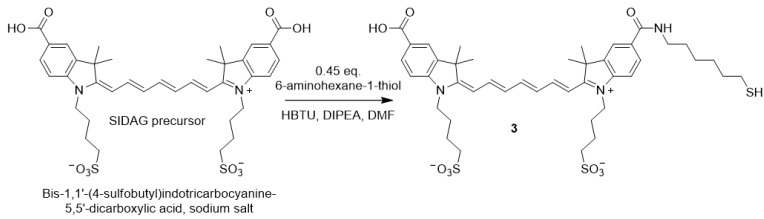
Cyanine derivative (SIDAG precursor [49]) and thiol-functionalized derivative **3** for coupling reactions via Michael addition.

Integrin α_v_β_3_, a transmembrane protein expressed on endothelial cells, and binds the Arg-Gly-Asp (RGD) triple peptide motif of extracellular matrix proteins. Growing malignant tumors continuously requires angiogenesis, and for this purpose the integrin α_v_β_3_ is overexpressed. As a result, α_v_β_3_ is preferentially expressed in tumor angiogenesis and is a potential target for AuNPs decorated with RGD peptides [47]. Therefore, ultra-small AuNPs (3 ± 2 nm) were synthesized by the Brust-Schiffrin-method [15] with thiol-PEG_3_-OH used as the stabilizing ligand and to achieve an increased biocompatibility. The AuNPs were further functionalized via ligand exchange with furan-protected maleimide-PEG_4_-thiol (Figure 1). Afterwards, the furan-maleimide-AuNPs **2a** were deprotected at 95 °C in DMSO for 2 h (**2b**), followed by the attachement with the thiol-functionalized α_v_β_3_-specific ligand thiol-c(RGDfK) **6** (Figure A1) and the thiol-functionalized fluorescent dye **3** (Figure A2) [43,51]. For the use in OI, the AuNPs were functionalized with **3**, which was performed at a ratio of 40:1 (AuNPs:dye). Using more NIR-dye resulted in the aggregation of the AuNPs as well as decreased fluorescence signals due to self-quenching. The purification of the AuNPs was performed via dialysis and size-exclusion chromatography. The AuNPs **7** and **8** were incubated with PBS, rat plasma, and cell media for at least 24 h at 37 °C, and no aggregation was found. Their stability was confirmed by UV/Vis spectroscopy and HPLC. The AuNP-RGD-NIR-dye **8** exhibited very low photobleaching (<3% within 24 h at 37 °C) and enabled biocompatible fluorescence in the optical window of tissue, with absorption of 600–800 nm (Abs_Max_ = 750 nm) and emission at 750–820 nm (Em_Max_ = 780 nm) (Figure A13).

**Figure A3:**
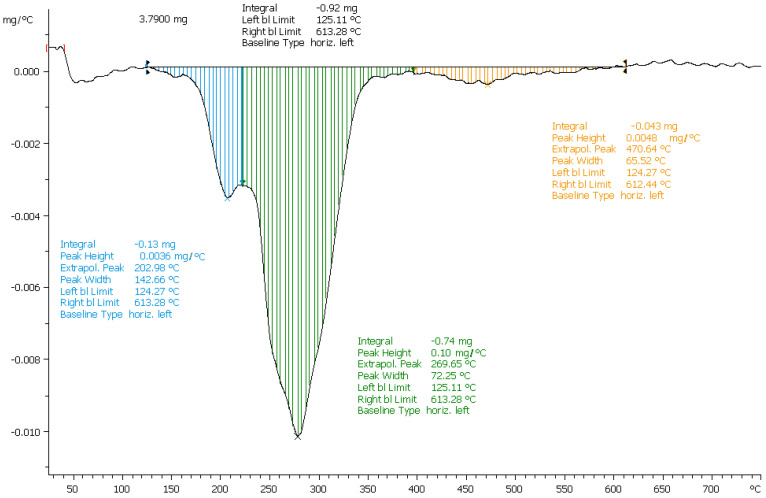
AuNP-PEG **1** with mass loss of ~24.27%.

**Figure A4:**
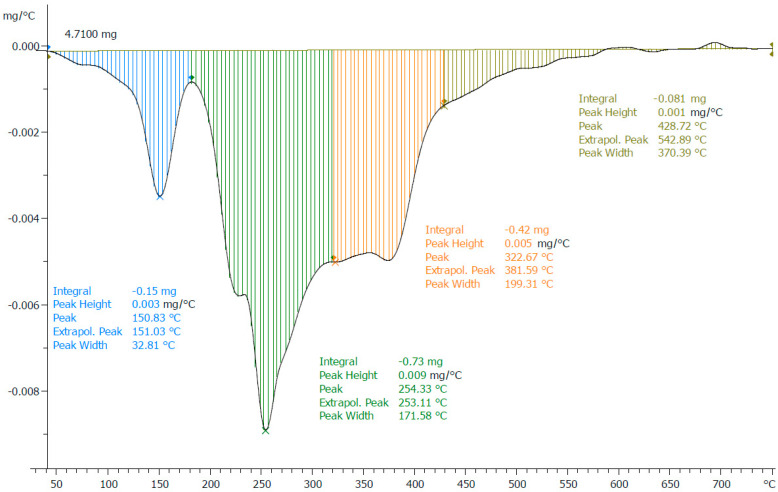
AuNP-maleimide **2a** with mass loss of 29.32%. Furan-PG (blue) is responsible for 3.18% mass loss. Maleimide-PEG_4_ accounts for a mass loss of 5% and 25% weight percent of all ligands.

**Figure A5:**
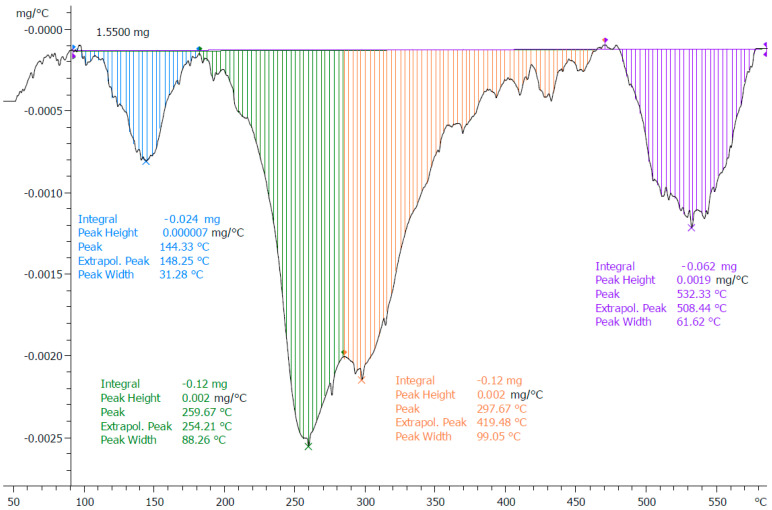
AuNP-RGD **7** with mass loss of 27.89%. Furan-PG (blue) is left with 2.11% mass loss. Instead, the RGD-ligands (purple) are responsible for 5.91% mass loss.

**Figure A6:**
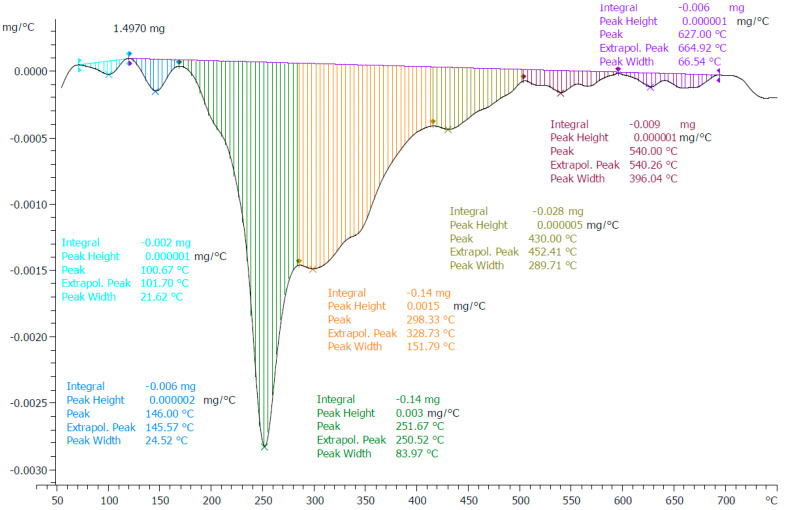
AuNP-RGD-NIR-dye **8** with mass loss of ~22.17%. The NIR-dye-ligands accounts for 0.46% mass loss.

**Figure A7:**
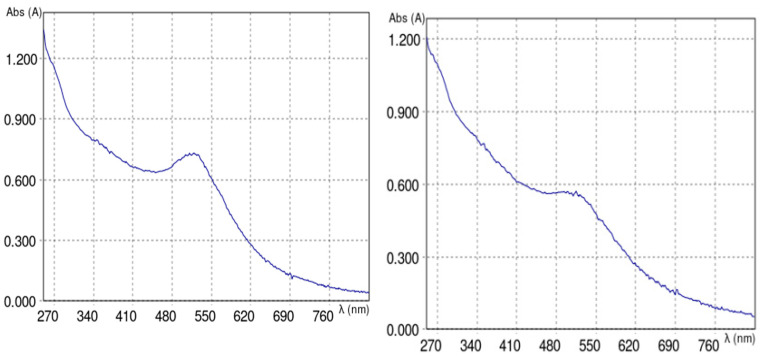
AuNP-PEG **1** (A_spr_ = 0.730/A_450_ = 0.633 give 1.15 → 3–4 nm) after dialysis with typical absorption maximum for ultrasmall AuNPs at 514 nm (**left**). AuNP-maleimide **2a** (**right**).

**Figure A8:**
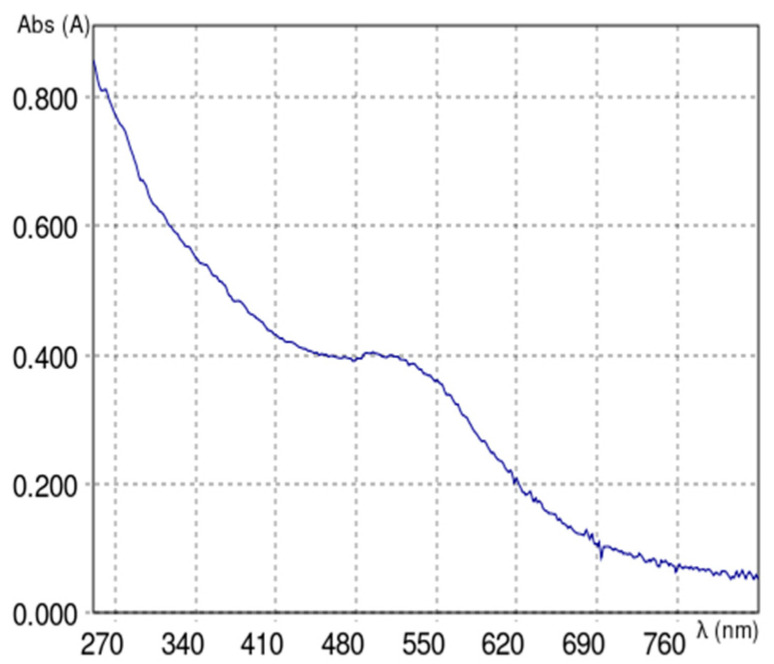
Absorption spectrum of AuNP-RGD **7** after dialysis (A_spr_ = 0.399/A_480_ = 0.389 give 1.02 → 3 nm).

**Figure A9:**
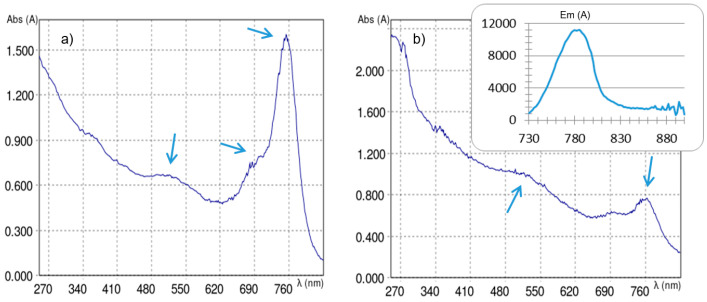
Absorption spectra of AuNP-RGD-NIR-dye **8** (**a**) after dialysis and (**b**) after additional Sephadex purification (A_spr_ = 0.668/A_480_ = 0.660 gives 1.01 → 3 nm) with three absorption maxima at 534, 690, and 750 nm. Insert in (**b**): Corresponding emission spectrum (excitation wavelength = 690 nm, emission = 740–840 nm, Em_Max_ = 790 nm).

**Figure A10:**
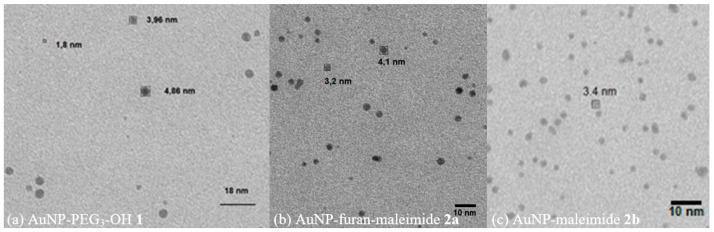
Transmission electron microscopy of (**a**) AuNP-PEG **1** (A = 10.53 nm^2^, Ø = 3.66 nm, n = 1000), (**b**) AuNPs **2a**, and (**c**) AuNPs **2b** (A = 9.21 nm^2^, Ø = 3.42 nm, n = 1860).

**Figure A11:**
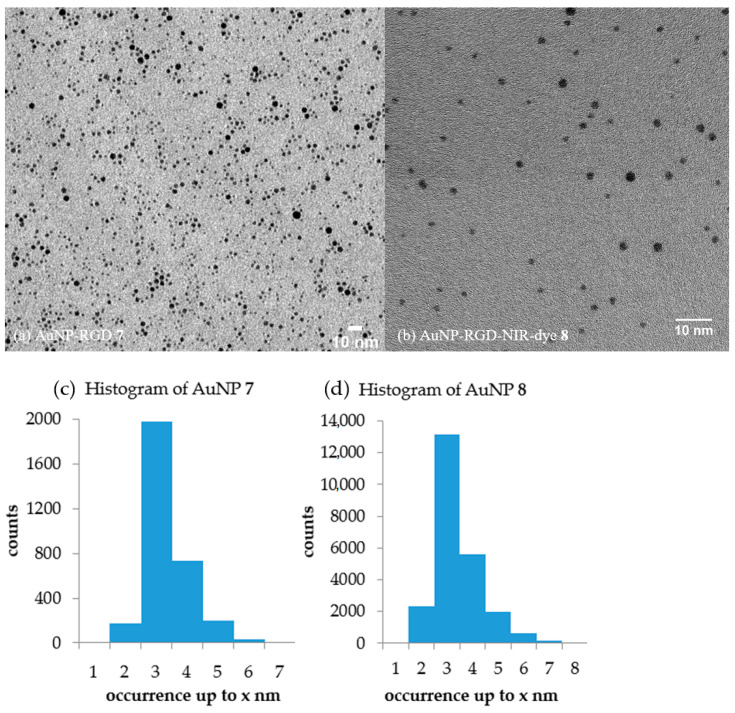
Transmission electron microscopy of (**a**) AuNP-RGD **7** (A = 6.64 nm^2^, Ø = (2.91 ± 2.27) nm, n = 3100) and (**b**) AuNP-RGD-NIR-dye **8** (A = 7.44 nm^2^, Ø = (3.08 ± 2.57) nm, n = 23,900) and the respective histograms (**c**) and (**d**) of the AuNP diameter distribution.

**Figure A12:**
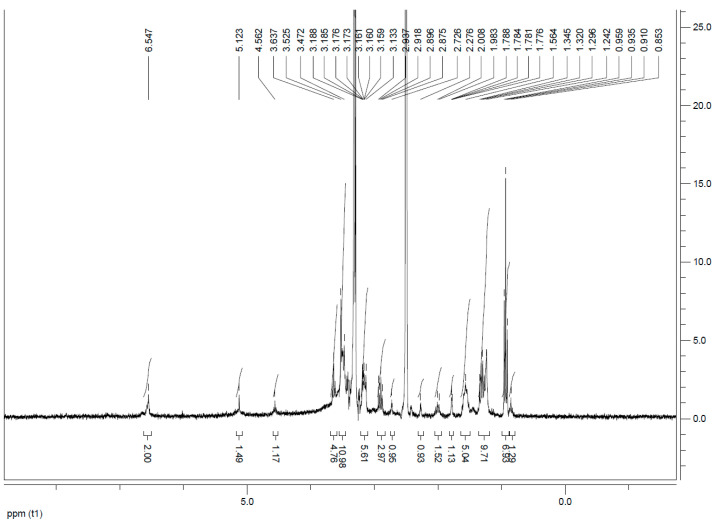
^1^H NMR spectrum of AuNPs **2a** in d_6_-DMSO with the characteristic signals of the furan-protected maleimide at 6.55, 5.12, and 4.56 ppm.

**Figure A13:**
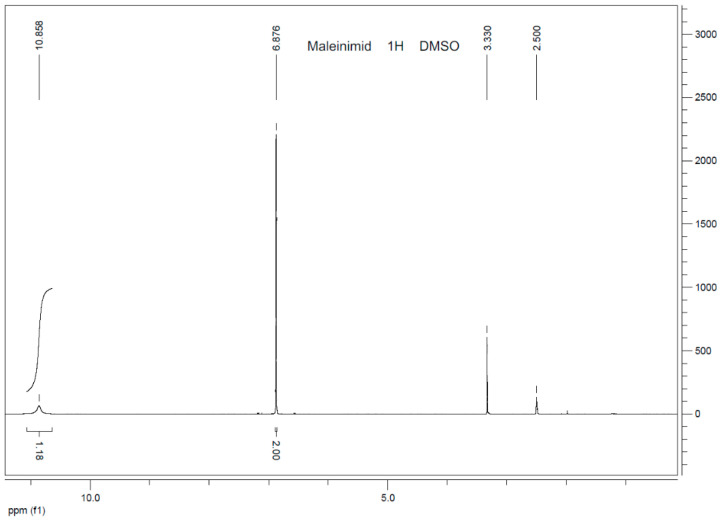
^1^H NMR spectrum of maleimide in d_6_-DMSO with the characteristic signal of the double bond at 6.88 ppm.

**Figure A14:**
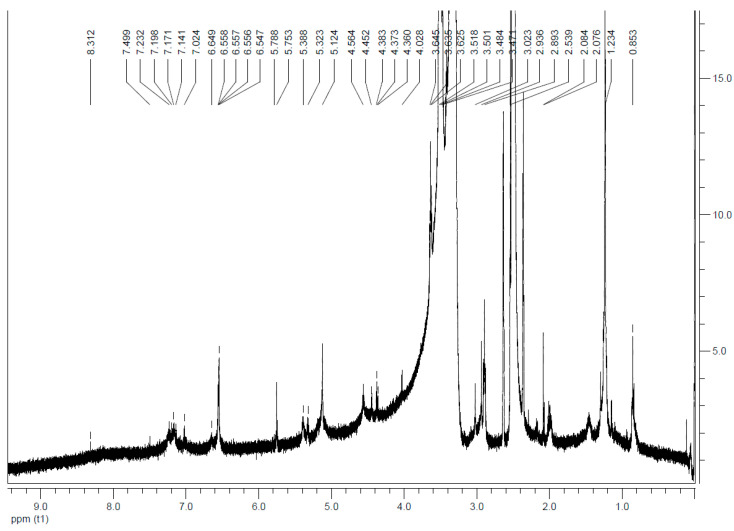
^1^H NMR spectrum of AuNP-RGD **7** in d_6_-DMSO: the characteristic signal of the double bond of the maleimide is faded; typical signals of peptide-bond-NH (7.50 ppm), Phe-CH_arom_ (7.23–7.14 ppm), Arg-C=NH (6.65 ppm), Arg-NH_2_ (5.78 ppm), Phe-CH (5.39 ppm), Asp-CH (5.32 ppm), Arg-CH (4.45 ppm), Lys-CH (4.38 ppm), Gly-CH_2_ (4.03 ppm), and residual furan-protected maleimide (6.55, 5.12 and 4.56 ppm).

**Figure A15:**
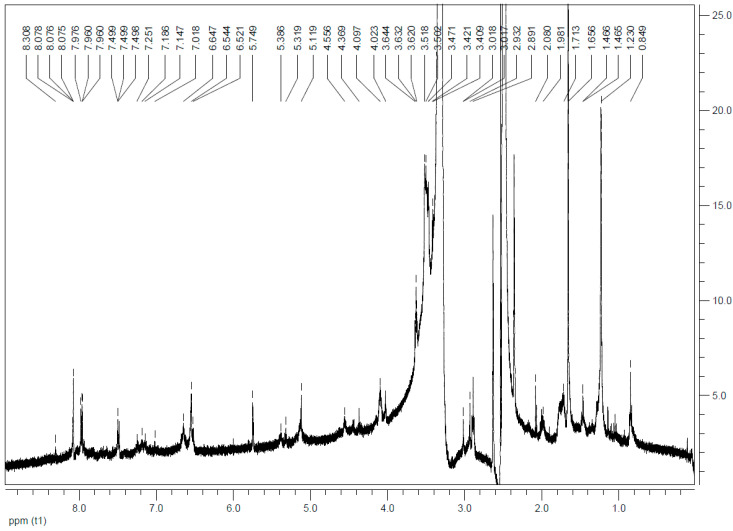
^1^H NMR spectrum of AuNP-RGD-NIR-dye **8** in d_6_-DMSO: typical signals of peptide-bond-NH (7.50 ppm), Phe-CH_arom_ (7.25–7.02 ppm), Arg-C=NH (6.65 ppm), Arg-NH_2_ (5.75 ppm), Phe-CH (5.39 ppm), Asp-CH (5.32 ppm), Arg-CH (4.56 ppm), Lys-CH (4.37 ppm), Gly-CH_2_ (4.02 ppm) and NIR-dye 8.08 (d, 2H, 6,6′-H), 8.05 (s, 2H, 4,4′-H), 7.96 (t, 2H, β,β’-H), 7.50 (m, 1H, δ-H), 7.3 (d, 2H, 7,7′-H), 6.65 (t, 2H, γ,γ’-H), 6.54 (d, 2H, α,α’-H), 4.09 (t, 4H, 2 × >NCH2) (compound **4a** in [51]) can be found.

The loss of mass of the AuNPs was determined by thermogravimetric analyses for each functionalization step. The measurements were compared between the product and educt to determine the additional loading of the respective functionalization step. Therefore, the difference in the loss of mass of product and educt gave the total mass of newly attached molecules. The mean number of newly attached molecules could be estimated by dividing the mass difference by the molar mass of the respective molecule. After knowing the number of the newly attached molecules, a formula from Zhu et al. was used to calculate the total molar mass of the AuNPs [42] (Table 1). A brief description of the synthesis and characterization can be found in the appendix. All AuNPs were fully characterized by thermogravimetric analysis (TGA) (Figure A3, Figure A4, Figure A5 and Figure A6), UV/Vis spectroscopy (Figure A7, Figure A8 and Figure A9), electron microscopy (EM) (Figure A10 and Figure A11), HPLC (Table 1), dynamic light scattering (DLS) (Table 2), and NMR (Figure A12, Figure A13, Figure A14 and Figure A15). The dynamic light scattering was performed on a Malvern Zetasizer ZS90. AuNPs were dissolved in distilled H_2_O at a concentration of 1 µg/mL. All values are the mean value of at least three different measurements. If the polydispersity index (PDI) is >0.5 the size by number is more relevant than the size by volume. The size by number is best comparable to the size measured by EM. The AuNPs could be stored in lyophilized form for >12 months at −20 °C without losing their integrity. In contrast, if stored in solution at room temperature, aggregation in form of precipitation occurred within weeks, especially for peptide- or NIR-decorated particles [33].

### 3.2. Cell Experiments

**Figure 2:**
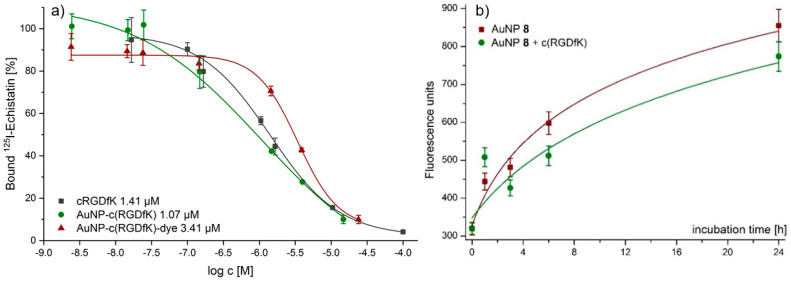
(**a**) Three representative IC_50_-curves from single competitive binding experiments. Shown is one curve with single IC_50_ value out of three values from three different experiments. (**b**) Cell uptake studies of AuNPs **8** in A431 cells at different time points obtained without blocking (red) and with a 10-fold excess of c(RGDfK) (green).

Several different IC_50_ values for RGD derivatives are already available in literature, ranging from 0.1 nM up to 6.7 µM. The main reason for the observed differences is the assay method used to determine the IC50 values. IC_50_ values of 0.1–1 nM can be found for RGD peptides having been determined by ELISA assays [45] and IC_50_ values around 20 nM were reported for solid-phase α_v_β_3_ binding assays for monomeric RGD derivatives [44]. Those IC_50_ values were derived by non-living experiments. Cell experiments are closer to in vivo condition. Therefore, for the AuNPs **7** and **8**, the α_v_β_3_-avidities were determined by competitive displacement experiments on α_v_β_3_-expressing A431 cells using ^125^I-Echistatin as α_v_β_3_-specific radioligand and competitor (Figure 2a). As internal reference, the RGD monomer c(RGDfK) was evaluated. With the evaluation of RGD derivatives by displacement experiments, IC_50_ values comparable with those in existing literature were found [48]. For c(RGDfK) an IC_50_ value of (1.75 ± 0.84) µM was found. The multi-RGD decoration at the surface of AuNP **7** led to a lower IC_50_ value of (1.07 ± 0.74) µM compared to the isolated RGD monomer. Further functionalization of the AuNPs with cyanine dye **3** led to a slightly higher IC_50_ value of (3.37 ± 0.73) µM for AuNP **8**.

Next, the cellular uptake of AuNPs **8** was evaluated on A431 cells at different time-points from 1–24 h. An at least partly receptor-specific uptake was found for the AuNPs (Figure 2b), as the cellular uptake of the dually modified particles could be partly blocked by pre-incubation using a 10-fold excess of c(RGDfK) 1 h before incubation with AuNPs.

**Figure A16:**
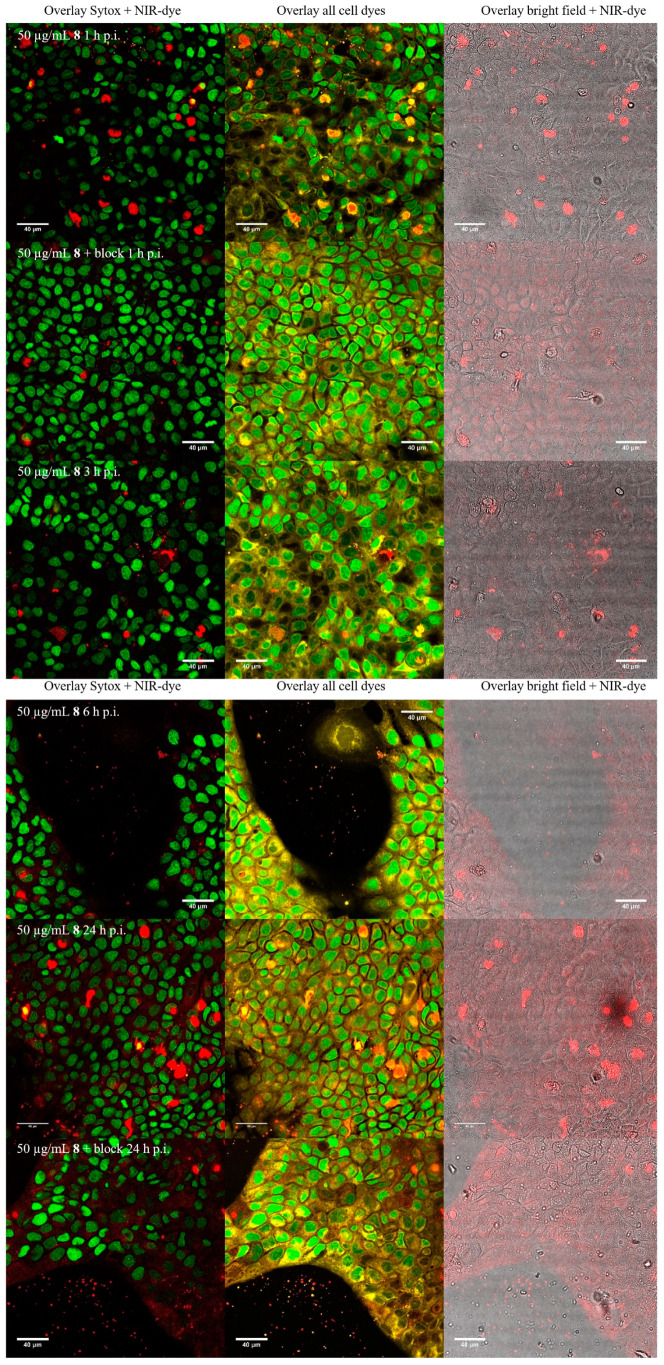
Fluorescence microscopy of A431-cells incubated with AuNP-RGD-NIR-dye **8** at same concentrations (50 µg/mL) at 1, 3, 6, and 24 h (from top to bottom). Left row overlays of Sytox Green and **8** are shown. Middle row overlays of Sytox, membrane orange and **8** are shown. Right row overlays of bright field and **8** are shown. The cell nuclei were stained with Sytox Green and the cytoplasma with membrane Orange.

Furthermore, the AuNPs **8** were tested for their behavior in vitro on A431 cells via fluorescence microscopy. First, the concentration for optimal microscopy was evaluated in a concentration range between 10–100 µmol/mL after an incubation time of 24 h. These initial cell studies revealed an optimized concentration of 50 µg/mL of AuNPs **8** for cell imaging. Next, via confocal fluorescence microscopy, the cell internalization, and the fate of the AuNPs were observed at different time points from 1–24 h (Figure A16). The confocal microscopy images revealed several findings: The number of observed fluorescence foci in the cells rises within the observation time from 1–24 h indicating an accumulation of AuNPs within the cell plasma. Further, these foci become bigger after 1 h of incubation. The AuNPs accumulated most probably in vesicles within the cells. At later time points, the vesicles appeared to become smaller containing less particles, whereas several new and much smaller foci showed up outside the cells. This is perhaps explained by the formation (and their later excretion) of vesicles containing the AuNPs. The conclusion that the mentioned dots represent vesicles is confirmed by the fact that the structures can be stained with membrane Orange, but not with the nuclei marker Sytox Green. This means that the vesicles should consist of excreted cell plasma compartments, underlining that the AuNPs are not accumulating within the cell nuclei. Moreover, the vesicles within in the cells showed a higher fluorescence signal for both membrane Orange and NIR dye.

### 3.3. Radiosensitizing Experiments

The developed AuNPs are useful for diagnosis but could also serve as therapeutic agents in form of radioactive [^198^Au]AuNPs or as radiosensitizer for X-ray irradiation. In this work, their ability as radiosensitizer was evaluated by irradiation of AuNP-incubated DNA and subsequent gel electrophoresis. AuNPs are effective secondary electron emitters when irradiated with X-rays due to their high photoelectric absorption [36] and their application as radiosensitizers in nuclear medicine is lively discussed [37,52,53]. If they are exaggerated by an external radiation source, Auger-Meitner electrons (AMEs) are emitted. This property was tested at the most prominent cell damage: double strand breaks (DSBs) of DNA. The induced radiation damage to pUC19 plasmid DNA was investigated as a function of dose and concentration of AuNPs. Indirectly induced single strand breaks (SSBs) were confirmed by using DMSO. DMSO can capture OH-radicals to form methanesulfinic acid (MSA) and is therefore a very prominent radical scavenger [54]. The concentration of DMSO is high enough to reach a scavenging effect for several weeks.

**Figure A17:**
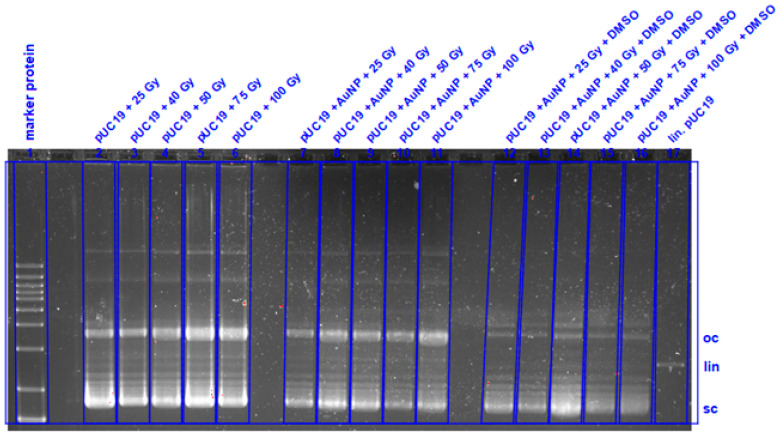
Evaluation of the irradiation experiments using X-ray in combination with AuNP **1** via gel electrophoresis. Left line is the marker protein. The lowest bands show intact sc-DNA, middle bands show lin-DNA with DSBs, and the highest band show oc-DNA with SSBs. The direction of elution is from top to bottom.

**Figure A18:**
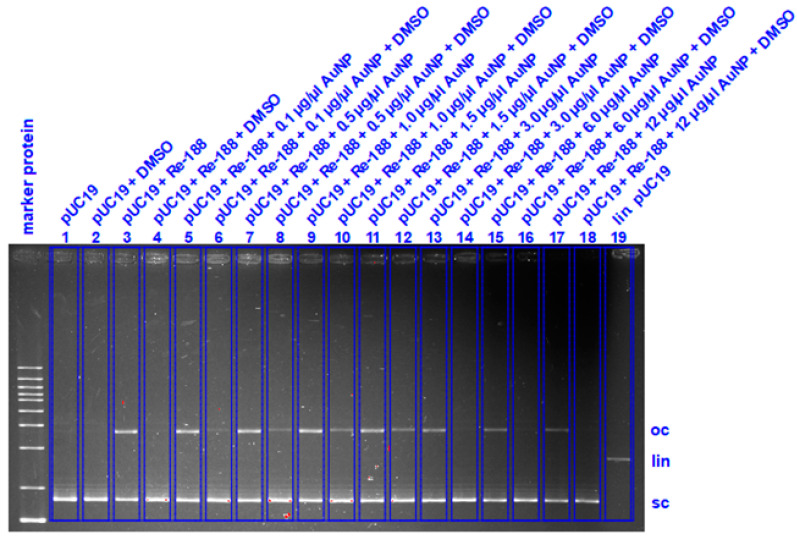
Evaluation of the irradiation experiments using Re-188 in combination with AuNPs **1** via gel electrophoresis. Left line is the marker protein. The lowest bands show intact sc-DNA, middle bands show lin-DNA with DSBs, and the highest band show oc-DNA with SSBs. The direction of elution is from top to bottom.

Two different AuNP derivatives, non-targeted AuNP-PEG **1** and targeted AuNP-RGD **7**, were tested for their radiosensitizing properties. The pUC19 plasmid DNA (280 kDa, 10 ng/µL per sample, BioLabs, New England) served as a biological model. A semi-quantitative analysis was performed to prove the therapeutic efficiency of the AuNPs. To distinguish between DNA damage caused directly (e.g., by AE) and indirectly (especially by OH-radicals), all experiments were repeated in the presence of DMSO (2 M, applied as radical scavenger). DNA damage was quantified by agarose gel electrophoresis and compared with 1 Kb Plus DNA ladder protein (Thermo Fisher) and linear plasmid derived from pUC19 by BamH1-kit. Three different conformation states were evaluated: Supercoiled (native form, sc), open circular (according to SSBs, oc), and linear (after DSBs, lin) (Figure 2). After irradiation, 10 µL samples were mixed with 1.25 µL 10 × BlueJuice gel loading buffer for tracking of DNA migration. After gel electrophoresis (2 h at 120 V, 400 mA, 120 W) the gels were stained with ethidium bromide and the relative fluorescence intensities of the fractions were calculated (BioRad Fluorescence Analyzer) (Figure A17 and Figure A18).

**Figure 3:**
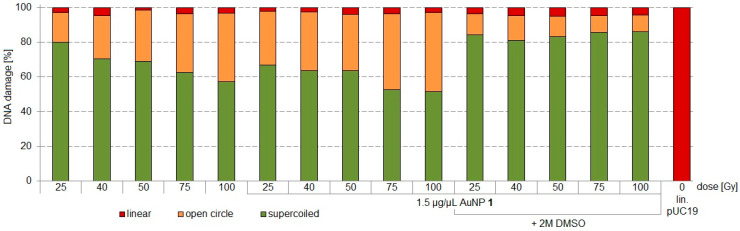
Irradiation experiments applying different doses of 25, 40, 50, 75, and 100 Gy for pUC19 only, pUC19 with AuNPs **1,** and pUC19 with AuNPs **1** in presence of 2 M DMSO. sc pUC19 is expressed as green bar, the extent of oc is expressed in orange, and the extent of lin plasmid in red.

In Figure 3, SSBs without AuNPs are found in form of oc-DNA of 17–40% between 25–100 Gy X-ray irradiation (Maxishot Y.TU, Yxlon). DNA incubated with AuNP **1** (1.5 µg/µL) showed oc-DNA of 31–46% between 25–100 Gy X-ray irradiation, indicating a 14% higher SSB induction at a dose of 25 Gy and 6% more SSBs at a dose of 100 Gy. At lower doses, the higher radiosensitizing effect of AuNP **1** was partially quenchable with DMSO to <10% oc-DNA. The radiosensitizing effect was highest at 25 Gy. DSBs were induced in every experiment to the same extent (3 ± 2%) and were not quenchable by DMSO. Therefore, no direct DNA damage was observable, but a higher indirect DNA damage at AuNP-incubated DNA was found.

**Figure 4:**
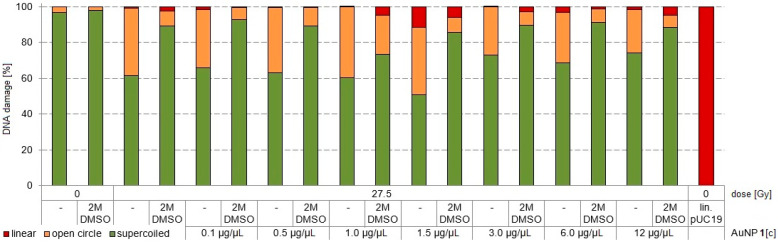
Irradiation experiments with 27.5 Gy Re-188 at different concentrations of AuNP-PEG **1** for pUC19 only, pUC19 with AuNPs and pUC19 with AuNPs in combination with DMSO. sc pUC19 is expressed as green bar, the extend of oc is expressed in orange and the extend of lin plasmid in red.

Next, the radiosensitizing effect at the same dose but different AuNP concentrations was evaluated (Figure 4). Further, we wanted to test whether it is possible to induce more DSBs by more production of AMEs, when the radiation source is in the direct vicinity of the AuNPs. For this experiment, Re-188-solution as inducer for radiosensitizing effects was chosen with its characteristic 2.12 MeV β^-^ emission and 155 keV γ-coemission. The highest effect for radiosensitizing was determined for 27.5 Gy as incubation dose. To reach this dose, 0.5 MBq Re-188 within 18 h incubation time in a 50 µL volume in a 1.5 mL Eppendorf vial were calculated using Formula 1. AuNP concentrations from 0.1–12.0 µg/µL were tested. Interestingly, an optimal radiosensitizing effect for AuNP-concentrations between 1.0–1.5 µg/µL was found. At these AuNP-concentrations the direct DNA damage in form of DSBs was 4.8–11.7%. This effect was completely quenchable by 2 M DMSO for Re-188 alone, meaning that there are only nascenting OH-radicals responsible for the DNA damage. In contrast, by the combination of Re-188 with AuNPs **1**, this effect was only partially quenchable by 2 M DMSO, meaning that additional AMEs from the AuNPs have a direct impact to the DNA damage (Figure A18). From these experiments it can be concluded that Re-188 in combination with AuNPs have a higher radiosensitizing effect than X-rays in combination with AuNPs. This effect has to be studied further.

(1) $$D\left(A,t\right)=S\cdot \frac{A\cdot T_{1/2}}{\ln\left(2\right)}\left(1-\exp(-\ln\left(2\right)\frac{t}{T_{1/2}})\right)$$

Formula (1) Calculation of ground dose in a 6-well-plate or Eppendorf vial for Re-188 by Geant4-simulation [55]. *D*: energy dose, *S*: S-value, *A*: activity, *T*_1/2_: half-live of radionuclide, *T*: irradiation time.

### 3.4. Colony Formation Assay (CFA)

**Figure 5:**
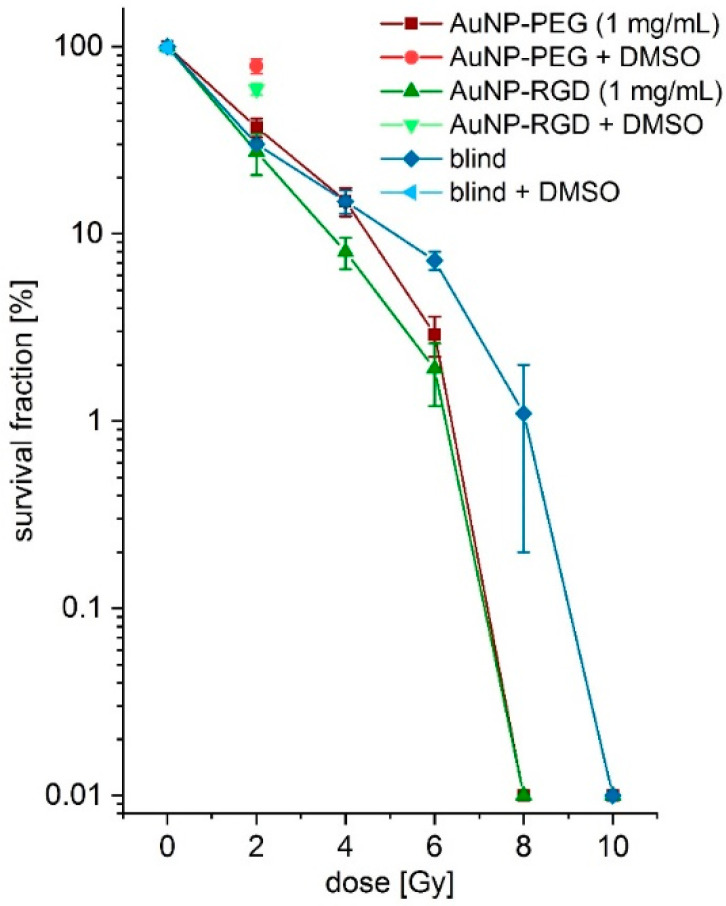
Survival fractions in logarithmic scale of the colony formation assays at different X-ray doses (0–10 Gy) with AuNPs **1** (red), AuNPs **7** (green), and without AuNPs (blue) incubation. Single colony formation assays (CFAs) with DMSO are shown at 2 Gy for AuNPs **1** (light red) and AuNPs **7** (light green) and at 0 Gy in absence of AuNP (light blue).

The radiosensitizing effect found for the DNA experiments was repeated in vitro by irradiation of AuNP-incubated and non-incubated A431 cells and subsequent CFA [52,53]. AuNPs within cell plasma are discussed to damage certain plasma compartments by additionally produced Auger-Meitner-electrons during X-ray irradiation [56]. This hypothesis was proven in a pilot experiment. First, AuNPs **1** were tested for cell toxicity with different concentrations on A431 cells by CFA. No cell toxicity was found up to a concentration of 1 mg/mL (2.9 µM) (Figure A21) comparable with the literature data [57,58,59]. Therefore, A431 cells were incubated with and without AuNPs **1** and **7** 24 h before irradiation. After incubation, the cells were washed and subsequently treated with doses of 0–10 Gy by X-ray irradiation. Afterwards, the cells were seeded for CFA. Indeed, a low difference in survival fractions for AuNP-incubated and non-incubated cells was found for a dose of 2 Gy (Figure 5). A slightly lower survival fraction for AuNP-incubated cells was found at 4 Gy for AuNP-RGD **7** (8.0 ± 1.5)% but not for AuNP-PEG **1** (15.0 ± 2.6)% compared to cells in absence of AuNPs (14.9 ± 2.1)%. A clearly much lower survival fraction at a dose of 6 Gy was found for AuNPs **7** (1.9 ± 0.7)% and AuNP **1** (2.9 ± 0.7)% compared to cells in absence of AuNPs (7.2 ± 0.8)% (Figure 5 and Figure A22), indicating a radiosensitizing effect. Further, at a dose of 8 Gy, no colony formation was found for AuNP-incubated cells. Furthermore, at a dose of 10 Gy, no colony formation was found for cells in absence of AuNPs. Interestingly, the specific AuNPs **7** showed a stronger effect at a dose of 4 and 6 Gy compared to non-specific AuNPs **1**. At a dose of 2 Gy, the cells were also incubated with 0.2 M DMSO as radical scavenger during irradiation. The factor 10 less DMSO concentration is important for cell life. These fractions showed lower cell damage for AuNP **7** (59.3 ± 4.3)% and much lower cell damage for AuNP **1** (78.9 ± 6.8). Non-incubated cells showed cell survival of (98.9 ± 5.2)% with 0.2 M DMSO at a dose of 0 Gy, indicating no toxic effects at these conditions.

### 3.5. In Vivo Experiments

**Figure 6:**
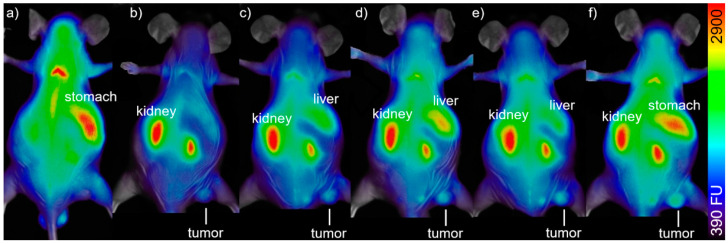
In vivo biodistribution of 50 µg/100 µL AuNPs **8** in a A431-tumorbearing mouse: (**a**) fluorescence before injection, (**b**) 4 h p.i., (**c**) 21 h p.i., (**d**) 28 h p.i., (**e**) 48 h p.i., and (**f**) 72 h p.i. Ratio tumor:muscle:liver:kidney/g = 4.4:1.0:0.6:5.4 (**e**).

**Figure 7:**
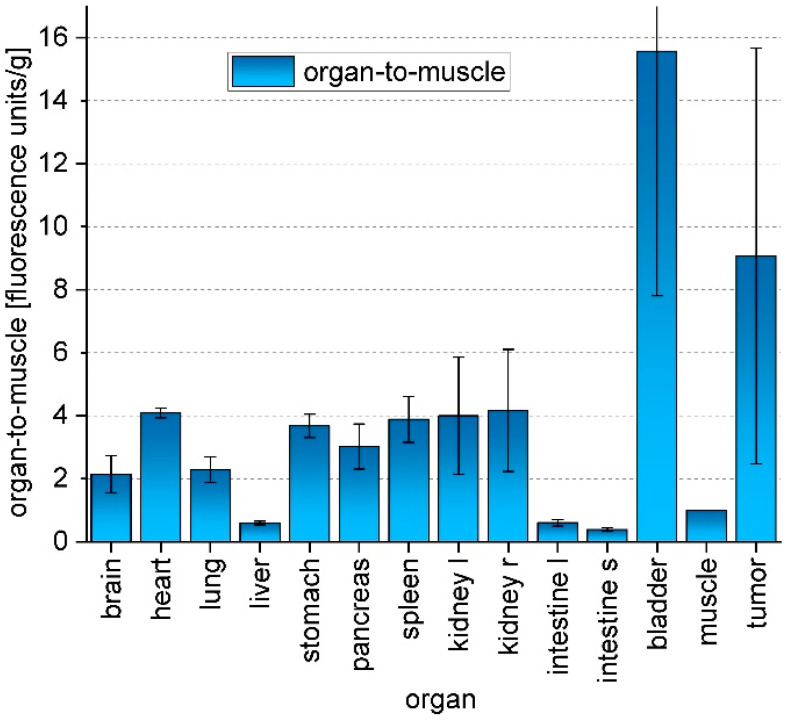
Biodistribution of AuNPs **8** at 72 h p.i. Values are given in measured fluorescence units per organ weight (n = 2).

In order to prove the concept of receptor-specific tumor accumulation of the peptide-functionalized particles and their suitability for optical imaging in vivo, two A431-tumor bearing mice were injected with AuNPs **8** according to previously established protocols [43]. In brief, the mice were injected with 75 µg AuNPs **8** in 100 µL sterile PBS. The injected amount of AuNPs **8** corresponded to 1.5 µg (1.75 nmol) of NIR-dye per animal as AuNPs **8** contain 2% dye (see Table 1). This amount is comparable to literature values of 1–50 µg/g for cyanine-dye conjugates for in vivo fluorescence imaging of tumors [10,49,60,61,62]. The mice were measured repeatedly over a period of up to 72 h post injection (p.i.) with a fluorescence imager (In Vivo Xtreme, Bruker) (Figure 6) by using 730 nm as excitation wavelength and 790 nm as emission wavelength. A very low background signal was found, which is assumed to derive from the chlorophyll-containing diet of the mice. After injection of AuNPs **8**, the background signal faded to the underground and a fast renal clearance of an excess of AuNPs **8** was observed in vivo. AuNPs **8** were found ex vivo in the collected urine and showed no degradation when analyzed by HPLC. The tumor-to-muscle ratio increased during the time of observation. The accumulation was measured by region-of-interest (ROI) interpretation (Figure A23), and after 72 h, an ex vivo biodistribution (Figure 7) was performed and compared with the obtained in vivo data. The accumulation values detected by in vivo measurements and biodistribution experiments were found to be comparable for tumor, muscle, and kidney but different for the liver, since in biodistribution experiments, the weight of the organs was also considered and the organs could be measured directly (Figure A24, Table A1), whereas in live imaging, the signal of the liver was found to be relatively lower, since the organ was measured from the back of the mice. However, the animal experiments revealed a higher accumulation of AuNPs **8** in the A431-tumor in comparison to muscle at 3–72 h post i.v. injection.

## 4. Discussion

Stable α_v_β_3_-specific AuNPs **7** were successfully synthesized with a slightly better avidity compared to the monomeric peptide ligand c(RGDfK). AuNPs additionally decorated with an NIR-dye had a slightly lower avidity as compared with the monomeric RGD-ligand, since in this case, a lower number of RGD ligands is located on the surface of the AuNPs (Table A1).

Via confocal fluorescence microscopy, the fate of AuNPs **8** on A431 cells was observed within 1–24 h. The AuNPs started to concentrate within the cell plasma but not within the nuclei as confirmed by confocal microscopy. Additionally, the formation of vesicles after 24 h and their excretion could be verified. The fluorescence signal started to concentrate in small dots within the cell plasma and in the extracellular medium those small foci could be observed too, indicating the excretion of the AuNPs in form of vesicles. This vesicle formation and excretion from cells within 24 h after incubation is perhaps a certain property of the AuNPs, when healthy tissue should expel therapeutic AuNPs. Within tumor tissue, the vesicles may stick in the interstitial cell region because of the lower blood supply and lower nutrition exchange with the surrounding tissues, presupposed that the AuNP would penetrate the deep tumor tissue.

**Figure A19:**
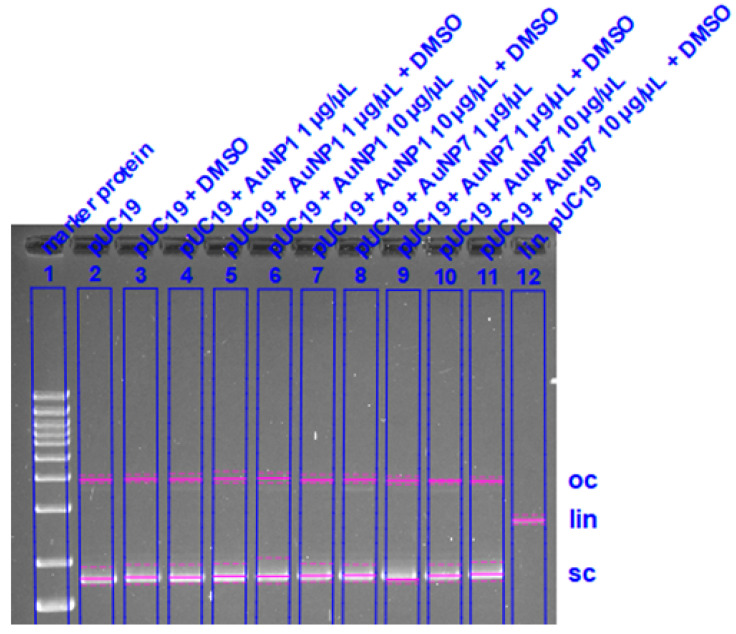
Evaluation of influence to DNA degradation of AuNP **1** and **7** at a concentration of 1 and 10 µg/mL with and without 2 M DMSO via gel electrophoresis. Left line is the marker protein. The lowest bands show intact sc-DNA, middle bands show lin-DNA with DSBs, and the highest band show oc-DNA with SSBs. The direction of elution is from top to bottom.

**Figure A20:**
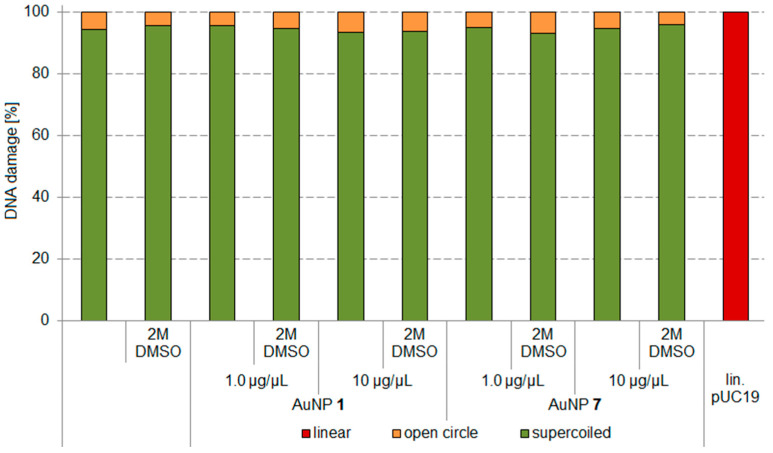
Influence of AuNPs **1** and **7** to the degradation of pUC19 DNA applying different concentrations of 0, 1, and 10 µg/µL with and without 2 M DMSO. sc pUC19 is expressed as green bar, the extent of oc is expressed in orange, and the extent of lin plasmid in red.

**Figure A21:**
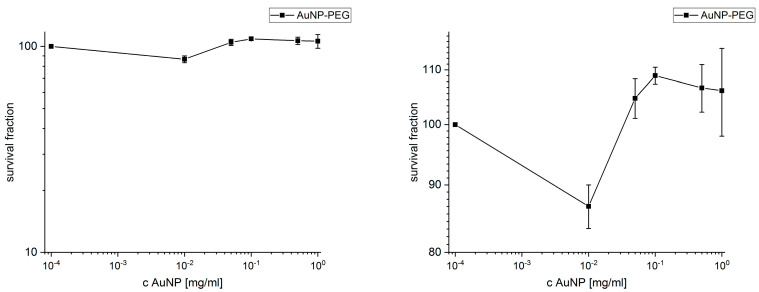
Toxicology assay for AuNP-PEG **1** with survival fraction 10–100% logarithmic scale (**left**) and 80–110% linear scale (**right**).

**Figure A22:**
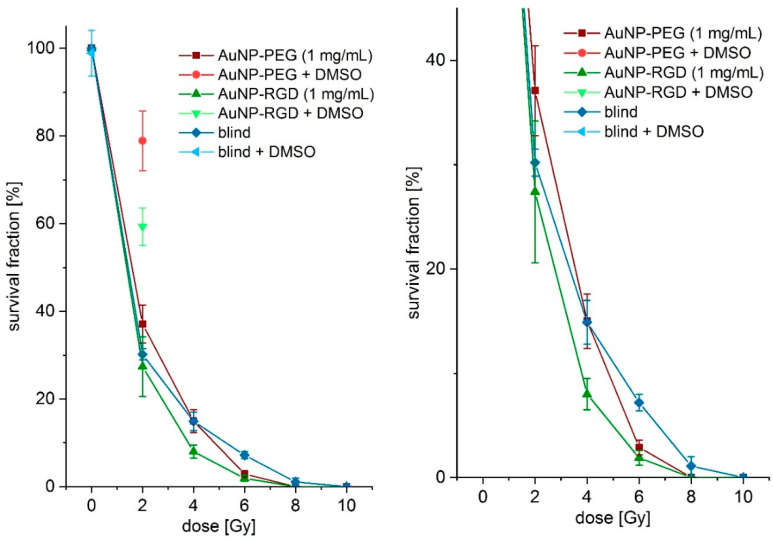
Colony formation assay with survival fraction 0–105% linear (**left**) and zoom 0–45% linear (**right**).

**Figure A23:**
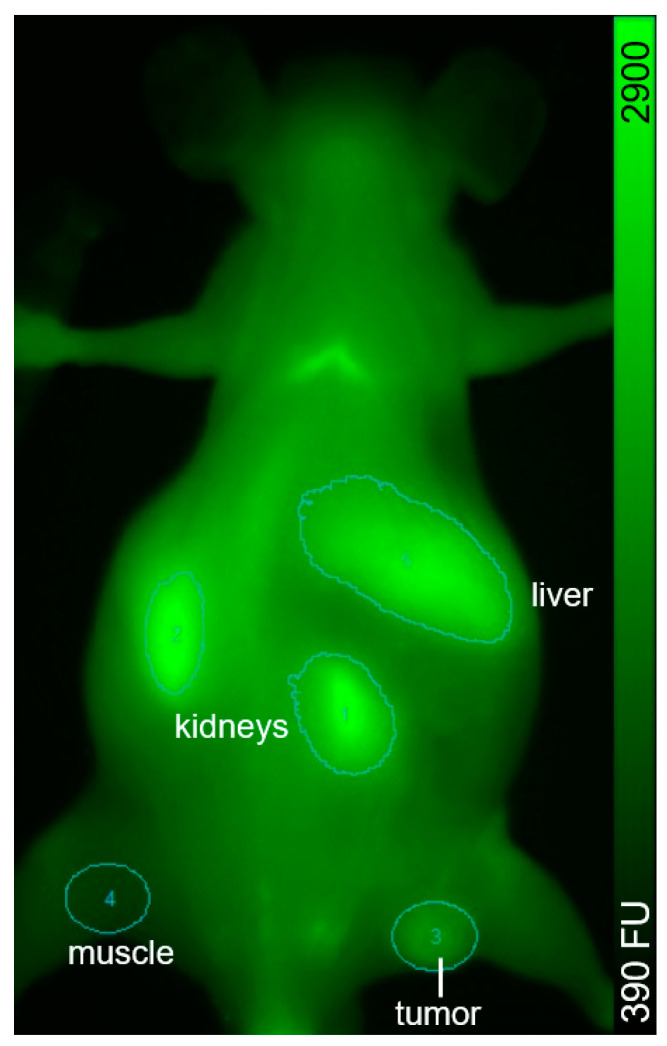
Regions of interest (ROIs) of organs during live imaging in vivo 48 h p.i. as example for AuNP-RGD-NIR-dye 8: ratio-to-muscle of kidneys 2.40 ± 0.12, tumor 1.47 ± 0.07, and liver 2.20 ± 0.11.

**Figure A24:**
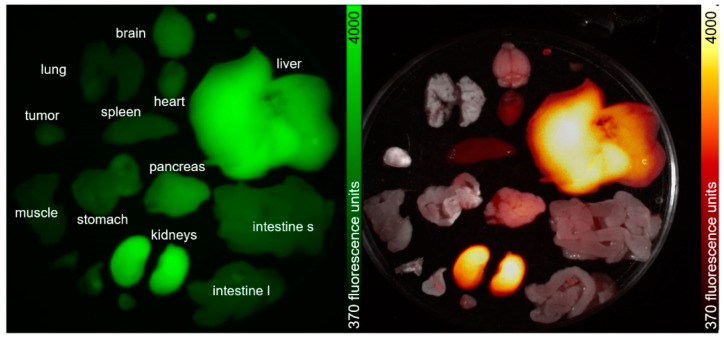
Ex vivo biodistribution of AuNP-RGD-NIR-dye 8 at A431-tumor-bearing mouse.

The activation of AuNPs by X-rays to emit AMEs was tested at pUC19 DNA incubated with AuNPs **1** [36,37,38]. No additional DSBs were found for all doses (25–100 Gy). Interestingly, additional SSBs (14%) in the presence of AuNP **1** were found at 25 Gy compared to non-AuNP-incubated DNA. Those SSBs were not inhibitable by DMSO, indicating the emission of AMEs from the AuNPs by external X-ray irradiation due to direct DNA damage without influence of OH-radicals. It is known that AuNPs can degrade DNA to little extend (1–2%) but does not increase significantly for longer time (weeks) and with organic solvents or no-neutral pH [63,64]. To further investigate this phenomenon, an experiment with AuNP **1** and **7** at concentrations of 1 and 10 µg/mL was performed to evaluate the impact of the AuNPs to the degradation of DNA (Figure A19 and Figure A20). The DNA degradation was not significantly different for all experimental conditions, and the formation of oc-DNA was at (5.48 ± 0.95)%. As for our experimental setting (2 h incubation, neutral pH), we can exclude that the >10% DNA damage is from the AuNP itself, but most prominent due to the induction of AMEs [65]. Further, an optimum for the AuNP concentration between 0.5–1.5 mg/mL was determined by incubation of DNA with Re-188 solution. The reason for this observation may be guessed by taking into account that at lower AuNP concentrations the number of additionally produced AMEs are too low for a visible DNA damage, whereas at higher concentrations, the AuNPs could shield the AMEs from a neighboring AuNP and Re-188. With DMSO as radical scavenger, the DNA damage was not completely quenchable.

The Auger–Meitner effect has a very short effective range of ~2 nm^3^ in vivo and is most effective when directly incorporated into the DNA [66]. Therefore, a nanoparticle not exceeding a diameter of 5 nm could in theory be an effective Auger-emitter in a volume of ~9 nm^3^ when activated with X-rays [36]. This is a very short range within a cell, which has a typical diameter of 120–200 µm. In vitro fluorescence microscopy confirmed (Figure A16) the accumulation of AuNPs in the cell plasma but not in the nuclei. For this reason, direct DNA damage can be excluded. Due to their behavior, the AuNPs could reach other cell compartments in the cell plasma that are also important for cell life [67]. Auger–Meitner electrons could then damage those compartments and the cell is about to die [56]. This hypothesis was evaluated in the following experiment: First, we carried out in vitro radiosensitizing experiments with AuNP-PEG **1** and AuNP-RGD **7** on A431 cells showing a similar effect as compared with literature data [37,38]. In the CFA experiments (Figure 5), AuNPs **1** and **7** did not have a much higher effect at a dose of 2 Gy compared to reference experiments in absence of AuNPs and had a slightly higher effect at 4 Gy compared to the untreated cells. AuNPs **7** had a higher effect at 6 Gy compared to AuNPs **1**. These findings could be explained by a faster accumulation of the targeted AuNPs **7** from the medium into the cell plasma compared to unspecific AuNPs **1** and other AuNP-species [43]. Perhaps bigger AuNPs could produce more Auger electrons or remain within the cells for a longer period of time, thereby allowing a higher effect to be observed. In addition, bigger gold nanorods (AuNRs) functionalized with RGD peptides could be an interesting Auger–Meitner emitter [68].

Initial in vivo experiments with AuNP-RGD-NIR-dye **8** showed a similar tumor accumulation and biodistribution within 72 h as compared to other NIR-dye-functionalized AuNPs [43]. In fluorescence live-imaging the organ-to-muscle ratios were 2.40 ± 0.12 (kidneys), 1.47 ± 0.07 (tumor), and 2.20 ± 0.11 (liver) (Figure A7). In biodistribution experiments, a significantly higher uptake in the tumor was found compared to the muscles (Figure 7). There was a relatively large difference in tumor uptake due to the different growth rate of the tumors. It is known that growing tumors have a high angiogenesis level and therefore a higher α_v_β_3_-integrine expression [47]. Hence, a higher uptake of RGD-functionalized AuNPs in a fast-growing tumor is explainable.

## 5. Conclusions

These initial results prove that the dually modified particles show potential as imaging tools for α_v_β_3_-expressing tumors in vivo via optical imaging. Especially for long-term observations of in vivo processes, the AuNPs could be useful. Since their accumulation is from slower nature (>1 d), the AuNPs cannot be used for short-term investigations such as Ga-68-PET. Further, the AuNPs could also serve as a tool for characterization of cell cycles and behavior by confocal fluorescence microscopy or live-cell microscopy. Interestingly, different modifications lead to different cell answer such as vesiculation and excretion. In vitro experiments for the assessment of the radiosensitizing ability of the AuNPs showed limited effects for the ultra-small AuNPs. However, in vivo, a major amount of AuNPs is excreted very fast renally. The rest of AuNPs accumulate in target organs and have a high retention there. Therefore, these AuNPs could serve as tool for radiotherapy as radiosensitizer or as activated [^198^Au]AuNPs. Further in vivo experiments for the determination of radiosensitizing effects for bigger AuNPs and AuNRs and also for [^198^Au]AuNPs are underway.

## Figures and Tables

**Table 1:** Calculated number of ligands and resulting molecular mass of the AuNPs and retention in HPLC.

Probe	Description	Number of Ligands	Molecular Mass [kDa]	Retention Time HPLC [min]
**1**	AuNP-PEG	500 PEG_3_-OH	345	6.48
**2a**	AuNP-PEG-maleimide	130 PEG_4_-maleimide	361	6.10
**7**	AuNP-RGD	60 RGD	405	5.98
**8**	AuNP-RGD-NIR-dye	~50 RGD, 10 NIR-dye	413	6.20

**Table 2:** Different expressions of the hydrodynamic radius of the AuNPs measured by DLS.

Probe	Size by Number [nm]	Size by Volume [nm]	Z-Averge [nm]	PDI
**1**	7.2	8.7	311	0.555
**2a**	14.1	13.6	209	0.456
**7**	33.8	32.6	713	0.883
**8**	53.8	59.8	174	0.403

## Data Availability

The data presented in this study are available on request from the corresponding author. The data are not publicly available due to institutional data protection.

## Appendix A. Organic Syntheses

c(RGDfK) peptide: The cyclic pentapeptide c(RGDfK) was synthesized in 0.2 mmol scale by solid-phase peptide synthesis on solid support using the standard Fmoc strategy on 250 mg H-Asp(*t*Bu)-2-chlortrityl-resin (loading: 0.8 mmol/g). For amino acid conjugation, 3.9 eq. HBTU (*N*,*N*,*N’*,*N’*-tetramethyl-*O*-(*1H*-benzotriazol-1-yl)uronium hexafluorophosphate), 4.0 eq. of Fmoc-protected amino acids, and 4.0 eq. of DIPEA were used in DMF as solvent. Coupling times were 30 min for each amino acid. After coupling of the last amino acid and Fmoc removal, the linear, protected peptide was cleaved from the resin using 1% TFA in CH_2_Cl_2_. The crude intermediate was isolated by evaporation of the volatile components of the mixture and then dissolved in dry DMF (120 mL). To this solution, DIPEA (64 µL, 0.70 mmol, 3.5 eq.) was added and the solution was cooled to 0–4 °C before DPPA (44 µL, 0.25 mmol, 1.25 eq.) was added. The mixture was allowed to warm to ambient temperature and reacted for 96 h until the cyclization was complete. The volatile components of the mixture were removed in vacuo, relyophilized in acetonitrile:water 1:1, and the residue was treated for 3 h with a mixture of TFA/TIS (triisopropylsilane) 97.5:2.5 (5 mL) to completely deprotect the peptide. After concentration in vacuo and precipitation of the product in cooled diethyl ether (25 mL), the crude product was obtained by centrifugation, washing with Et_2_O (5 mL) twice, and drying of the obtained solid. Purification of the product was performed by semi-preparative HPLC (*t*_R_ = 3.75 min; gradient: 0–10% MeCN in H_2_O with 0.1% formic acid in 6 min at 4 mL/min). The product was obtained after lyophilization as a colorless solid in 67% overall yield (81 mg, 134 mmol). MALDI-MS (calculated): *m*/*z* = 604.02 [M + H]^+^ (603.31), 626.32 [M + Na]^+^ (626.30), 642.45 [M + K]^+^ (642.28). Analytic HPLC: *t*_R_ = 1.61 min.

Thio-acetate-modified c(RGDfK) derivative **9** (Figure A1): To a solution of c(RGDfK) (5.6 mg, 9.28 µmol) in 0.15 M Sørensen phosphate buffer (0.5 mL, pH 7.2), a solution of 2,5-dioxopyrrolidin-1-yl-4-(acetylthio)butanoate (2.53 mg, 9.74 µmol) in MeCN:buffer v:v = 1:1 (0.3 mL) was added. After stirring the reaction mixture for 10 min at ambient temperature, the reaction was completed. The product was purified by semipreparative HPLC and obtained after lyophilization as a colorless solid with 85% yield (5.9 mg, 7.89 µmol). MALDI-MS (calculated): *m*/*z* = 748.06 [M + H]^+^ (747.34), 778.06 [M + Na]^+^, 786.07 [M + K]^+^. Analytic HPLC: *t*_R_ = 1.71 min.

Thiol-modified c(RGDfK) derivative **6** (Figure A1): To a solution of **9** (5.7 mg, 7.63 µmol) in dry MeOH (2.5 mL), a solution of 1 M NaOH in EtOH (7.63 µL) was added. After 45 min reaction at ambient temperature, 1 M HCl (19.83 µL) was added, and the reaction was completed after 5 min at ambient temperature. H_2_O (2.5 mL) was added for quenching and the product was lyophilized to generate the stable thiol **6** as a colorless solid with 84% yield (4.5 mg, 6.41 µmol). MALDI-MS (calculated): *m*/*z* = 706.18 [M + H]^+^ (705.33), 728.19 [M + Na]^+^, 748.26 [M + K]^+^. Analytic HPLC: *t*_R_ = 1.63 min.

Thiol-hexyl-cyanine **3** (Figure A2): Bis-1,1′-(4-sulfobutyl)indotricarbocyanine-5,5′-dicarboxylic acid sodium salt (SIDAG precursor) (50 mg, 68 µmol, Chess, Mannheim) was dissolved in DMF (6 mL). Thiol-functionalization was performed according to Licha et al. [69]. In brief, HBTU (10.2 mg, 27 µmol) and 6-aminohexane-1-thiol 5.3 mg, 31 µmol) were added and the solution was cooled to 0 °C. Then, DIPEA (18 µL, 102 µmol) was added and the reaction was stirred for 3 h at ambient temperature in the dark. Et_2_O (50 mL) was added to the solution and centrifuged. The Et_2_O was decanted and the residue was washed 3 × with Et_2_O, dried, and stored at −20 °C. The resulting green powder (54 mg) was used without further purification. The successful conjugation of 6-aminohexane-1-thiol was verified by Ellmans reagent and comparison of UV/Vis spectra at 412 nm. The purity was verified by HPLC (>80%). MALDI-MS (calculated): *m*/*z* = 856.066 [M + H]^+^ (855.11).

General procedure to prepare PEGylated AuNP **1**: Briefly, hydrogen tetrachloroaurate(III) trihydrate (560 mg, 1.44 mmol, ≥99.9% trace metal basis) was dissolved in 30 mL of tracepure water resulting in a bright yellow solution and then extracted by mixing with 300 mL of a tetraoctylammonium bromide (TOABr, 1011 mg, 1.85 mmol) toluene solution. The contents were stirred vigorously for 20 min at room temperature to facilitate the phase transfer of the Au(III) into the toluene layer, which resulted in the organic layer turning to a dark orange color and the aqueous layer becoming clear colorless. If the aqueous layer was not colorless, further TOABr (50 mg, 0.09 mmol) and toluene (100 mL) were added. After complete phase transfer, the aqueous layer was removed. The organic layer was dried with MgSO_4_ and filtered to remove excess of water. The solution was cooled to 0 °C in an ice bath. Then, 2.7 eq. of freshly prepared HO-PEG_3_-thiol (640 mg, 3.85 mmol) in 20 mL of dichloromethane was added and allowed to stir until the orange solution faded to colorless (~1 h). A fresh solution of tetrabutylammonium borohydride (TBABH) (3659 mg, 14.22 mmol) in 20 mL dichloromethane was then added to the rapidly stirring toluene solution over 5 s. The solution turned dark black instantly. The PEG-AuNP **1** started to precipitate from toluene after 1 h. After stirring the mixture for 16 h from 0 °C to 20 °C, 50 mL of tracepure water were added under slow stirring to extract the PEGylated AuNPs for 120 min. The organic layer was decanted, and the aqueous layer was washed alternatingly with 3 × 50 mL toluene/5 mL MeCN and 3 × 50 mL toluene/5 mL isopropanol. The black aqueous layer was transferred into a visking cellulose dialysis tube (molecular cut-off 14000 Da) with 3 × 10 mL tracepure water, and dialysis was performed in 3 × 10 L of distilled water for 1 h, 2.5 h, and 16 h. Afterwards, the AuNP **1** was lyophilized to yield 407 mg (44%) of black powder. These PEGylated AuNPs are relatively small (3.0 ± 2.0 nm), exhibit excellent stability, and can be repeatedly dried and dissolved in water.

General procedure for the preparation of furan-protected maleimide AuNP **2a**: The preparation of AuNP **2a** was performed by a place-exchange reaction of a freshly prepared furan-masked maleimide-PEG-thiol ligand with the PEGylated AuNPs. Maleimide-PEG-thiol ligand (120 mg, 0.34 mmol) was dissolved in 6 mL 1:1 tracepure H_2_O:MeOH and was added to PEG-AuNP **1** (300 mg) in 30 mL tracepure H_2_O and stirred for 90 min. A 1:1 mole ratio of maleimide ligand to PEG ligand is crucial. The mixed ligand AuNP **2a** sample was then purified by dialysis. Yield: 313 mg (99%).

General procedure of the Michael addition reaction: 20 mg of AuNP **2a** were dissolved in dry DMSO (3 mL) under argon atmosphere and stirred for 2 h at 95 °C to remove the furan-protecting group. Afterwards, the resulting AuNP **2b** solution was cooled to below 30 °C and 1 mg of thiol-functionalized c(RGDfK) peptide ligand **6** in dry DMSO (0.5 mL) was added under argon atmosphere. After 1 h at ambient temperature, 0.5 mg of dye-thiol **3** (0.58 µmol) in dry DMSO (0.5 mL) were added (Figure A2), and the mixture was stirred for 1 h. Then, tracepure water (26 mL) was added (>13% DMSO in water) and dialysis was performed against 10 L distilled water for 16 h. Afterwards, the resulting AuNPs were lyophilized.

## Appendix B. Characterization of AuNPs

Following the protocol from Zhu et al. [42] we used their formula to calculate the amount of gold atoms per nanoparticle. Since our nanoparticles have a mean diameter of ~3.5 nm, the calculated amount of gold atoms is ~1325 Au atoms per nanoparticle. This accounts for a molecular weight of Au 260,980 g/mol. Using TGA we found a mass loss for AuNP **2a** corresponding to a molecular weight of 360,611 g/mol with 370 PEG_3_-OH ligands and 130 PEG_4_-maleimide ligands.

### Appendix B.1. Determination of the Quantity of Ligands on the Surface of the AuNPs via Thermogravimetric Analysis

The thermogravimetric analyses were performed with a Mettler Toledo TGA/SDTA851^e^. AuNPs (2–5 mg) were weighed into 70-µL-aluminum oxide crucibles (Mettler Toledo, Gießen, Germany) and heated from 25–750 °C (10 K/min) in a N_2_ or CO_2_ stream (30 mL/min). The loading of the different AuNPs is shown in Table 1 and was calculated by the different mass losses, which increase the more the AuNPs are functionalized. Therefore, the quantity of the different ligands per particle can be calculated.

- The mass loss of the AuNP-PEG **1** was 24.27%. This accounts for ~500 PEG ligands at the AuNP surface. M ~ 345 kDA.
- The mass loss of AuNP-PEG-maleimide **2a** was 29.32%. The difference of 5.02% accounts for ~130 PEG-maleimide ligands. M~361 kDa.
- The mass loss of AuNP-RGD **7** was 27.89% and the RGD accounts for 5.91% mass loss (60 RGD ligands per AuNP). Hence, we can calculate the molar mass for example for AuNP-RGD **7** to be ~405 kDa.
- Further, the AuNP-RGD-NIR-dye **8** contained around 10 NIR-dye ligands.

### Appendix B.2. UV/Vis Spectra and Size Determination

UV/Vis measurements were performed with an Eppendorf BioSpektrometer Kinetic. We measured the absorption of the AuNPs at a concentration of 1 µg/mL to estimate the particle size before performing electron microscopy. The absorption at the surface-plasmon resonance (maximum) divided by the absorption at 450 nm (minimum) gives a factor that can be compared with tables from literature [70]. Emission scans were performed on a Tecan infinite M200 with excitation wavelength of 690 nm.

### Appendix B.3. Electron Microscopy

AuNP samples were diluted in deionized water at convenience (fade red solution), particles adsorbed onto glow-discharged carbon-coated EM-grids and directly observed by TEM (Zeiss EM912, Carl Zeiss Oberkochen or Titan 80-300, FEI Company, HZDR). Images were digitally registered via a CCD camera (Sharp eye, TRS, Moorenweiss or US1000 der Firma Gatan). The number and size of particles was measured by FIJI software (v1.50e).

## Appendix C. Cell Experiments

### Fluorescence Microscopy In Vitro

Initial cell studies revealed an optimized concentration of 50 µg/mL of AuNP-RGD-NIR-dye **8** for cell imaging. Via confocal fluorescence microscopy, the cell internalization of the particles, vesicle formation, and excretion were verified after 1, 3, 6, and 24 h (Figure A16). The cells start to excrete AuNPs after 3 h. The particles are located in the cytoplasm and not inside the nuclei. Fluorescence microscopy was performed on a Leica TCS SP8 confocal microscope with lasers at λ = 488 nm (Sytox), 552 nm (memOrange), and 638 nm (NIR-dye).

## Appendix D. Animal Experiments

**Table A1:** Biodistribution of AuNP-RGD-NIR-dye 8 in A431 tumor-bearing mice. MFU = Mean fluorescence units.

Organ	Weight [g]	MFU/g	Ratio-to-Muscle/g
pancreas	0.387	5089	3.53
kidney left	0.383	7651	5.31
kidney right	0.384	7975	5.54
heart	0.226	5730	3.98
lung	0.236	3709	2.58
muscle	0.558	1440	1.00
brain	0.417	3690	2.56
intestine large	1.096	983	0.68
spleen	0.228	4857	3.37
stomach	0.252	4923	3.42
intestine small	2.537	499	0.35
liver	3.215	927	0.64
gall			
bladder	0.034	30329	21.06
tumor	0.148	6346	4.41
